# Identification of Novel Compounds That Bind to the HGF β-Chain In Silico, Verification by Molecular Mechanics and Quantum Mechanics, and Validation of Their HGF Inhibitory Activity In Vitro

**DOI:** 10.3390/molecules30081801

**Published:** 2025-04-17

**Authors:** Ko Suzuki, Keitaro Inoue, Ryota Namiguchi, Seiya Morita, Suzuho Hayakawa, Mikuri Yokota, Katsuya Sakai, Kunio Matsumoto, Shunsuke Aoki

**Affiliations:** 1 Department of Bioscience and Bioinformatics, Graduate School of Computer Science and Systems Engineering, Kyushu Institute of Technology, Iizuka 820-8502, Japan; 2 Division of Tumor Dynamics and Regulation, Cancer Research Institute, Kanazawa University, Kanazawa 920-1192, Japan; 3 WPI-Nano Life Science Institute, Kanazawa University, Kanazawa 920-1192, Japan

**Keywords:** hepatocyte growth factor (HGF), SBDS, molecular docking, MDS, fragment molecular orbital, Met

## Abstract

The development of small-molecule drugs targeting growth factors for cancer therapy remains a significant challenge, with only limited successful cases. We attempted to identify hepatocyte growth factor (HGF) inhibitors as novel anti-cancer small-molecule drugs. To identify compounds that bind to the β-chain of HGF and inhibit signaling through HGF and its receptor Met interaction, we performed a hierarchical in silico drug screen using a three-dimensional compound structure library (Chembridge, 154,118 compounds). We experimentally tested whether 10 compounds selected as candidates for novel anticancer agents exhibit inhibition of HGF activity. Compounds **6** and **7** potently inhibited Met phosphorylation in the human EHEMES-1 cell line, with IC_50_ values of 20.4 and 11.9 μM, respectively. Molecular dynamics simulations of the Compound **6**/**7**–HGF β-chain complex structures suggest that Compounds **6** and **7** stably bind to the interface pocket of the HGF β-chain. MM-PBSA, MM-GBSA, and FMO analyses identified crucial amino acid residues for inhibition against the HGF β-chain. By interfering with the HGF/Met interaction, these compounds may attenuate downstream signaling pathways involved in cancer cell proliferation and metastasis. Further optimization and comprehensive evaluations are necessary to advance these compounds toward clinical application in cancer therapy.

## 1. Introduction

Hepatocyte growth factor (HGF) was initially cloned as a potent mitogenic protein for hepatocytes. However, it was later discovered that HGF also induces a variety of biological responses in epithelial cells, including proliferation, migration, survival, and morphological changes [1]. These effects are mediated by the activation of c-Met, a single-pass transmembrane receptor tyrosine kinase. Upon HGF binding, the Met receptor undergoes dimerization and autophosphorylation of its tyrosine kinase domain, which initiates a cascade of intracellular signaling pathways [2]. The HGF/Met signaling pathway plays a crucial role in promoting cancer cell invasion and metastasis, and it is also involved in the development of drug resistance in certain tumor cells [3]. Consequently, inhibitors targeting the HGF/Met signaling pathway have emerged as promising candidates for cancer therapy.

Structurally, HGF consists of six domains: the N-terminal (N) domain, four kringle (K) domains, and the serine protease homology (SPH) domain. It is secreted as an inactive single-chain precursor and is activated by proteolytic cleavage into two chains: the α-chain (NK4) and β-chain (SPH), which are linked by a disulfide bridge. Studies have shown that HGF binds to the extracellular sema domain of c-Met via two distinct binding sites: the K1 domain in the α-chain and the SPH domain [4]. Mutagenesis studies targeting a specific pocket structure within the Met-binding interface of the SPH domain have demonstrated that this pocket is essential for both binding to Met and activating the HGF/Met signaling pathway [5].

Several pharmaceutical companies have developed HGF-neutralizing monoclonal antibodies, and clinical trials are ongoing to evaluate their potential as anticancer agents, such as ficlatuzumab [6,7]. However, the high molecular weight of monoclonal antibodies presents significant challenges for drug delivery, and the complex manufacturing process contributes to their high cost. In contrast, there are currently no small-molecule compounds targeting the HGF/Met interaction in clinical development.

Previously, using an in silico approach, we identified a small molecule that binds to the HGF K1 domain and demonstrated its pharmacological activity in cell-based in vitro experiments [8]. This finding suggested that small molecules could potentially disrupt protein–protein interactions within the HGF/Met signaling pathway. In this study, we conducted the virtual screening of a compound library using structure-based drug screening (SBDS) to target the SPH domain of HGF. This led to the identification of two novel small-molecule compounds, Compound **6** and Compound **7**, which effectively inhibit the HGF/Met signaling pathway in cells.

## 2. Results and Discussion

### 2.1. Interface Pocket in the HGF β-Chain That Binds to the Met Sema Domain

**Figure 1:**
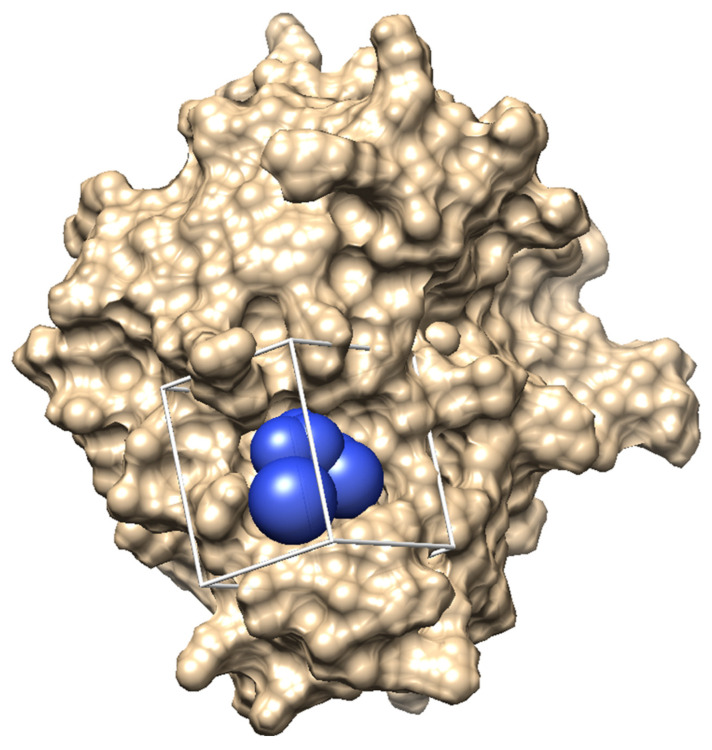
The pocket structure in the HGF β-chain serves as the binding interface with the Met. The blue spheres are virtual atom spheres set up for compound binding simulations using DOCK. The cube structure is a visualization of the compound search space using DOCK.

To target the HGF/Met interaction, the structure of the β-chain of hepatocyte growth factor (HGF) was used for the SBDS approach. The crystal structure data of the HGF β-chain (PDBID 4K3J, resolution: 2.80 Å) were obtained from the RCSB Protein Data Bank (PDB) [9]. The Met sema domain, onartuzumab, and ligand (NAG) were removed from the crystal structure data (4K3J). Hydrogen atoms and partial charges were added to the HGF β-chain structure, and energy minimization calculations were performed. We performed in silico SBDS targeting the pocket structure in the HGF β-chain that serves as the binding interface with the Met sema domain (Figure 1). 

### 2.2. In Silico Exploration of the HGF β-Chain-Binding Compounds

**Figure 2:**
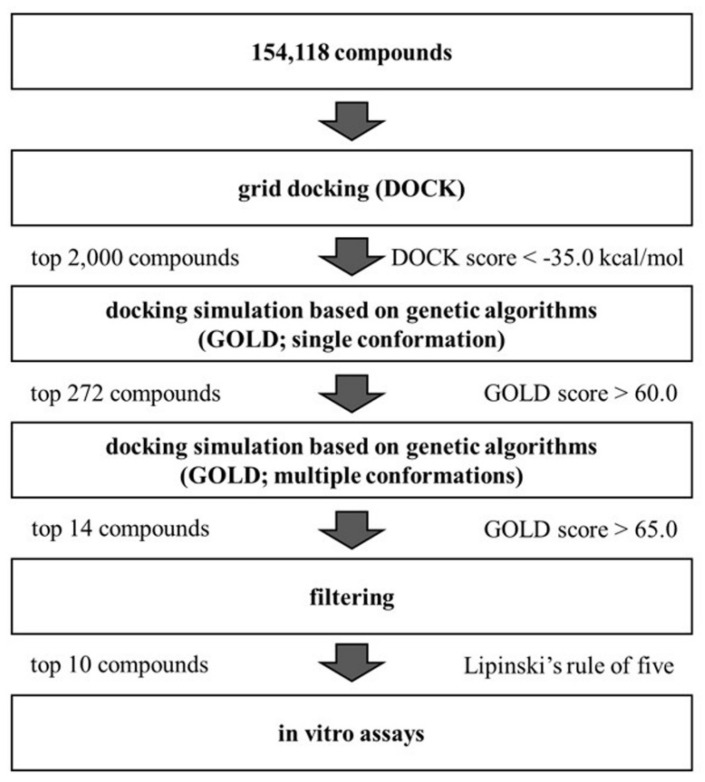
Hierarchical screening pathway for identification of the HGF β-chain inhibitors.

A three-step hierarchical screening using UCSF DOCK 6 [10] and GOLD 5. 2. 2 [11] was performed on a 3D structural library containing 154,118 compounds (Figure 2). In the first screening, rigid-body docking using DOCK was performed to select 2000 compounds with a predicted binding free energy lower than −35.0 [kcal/mol]. In the second screening, GOLD, a genetic algorithm-based docking simulation tool, was used to select 272 compounds with GOLD scores higher than 60. We generated up to 10 different conformations for each compound with GOLD scores higher than 60 and performed docking simulations with GOLD to accommodate more flexibility in compound structures. For the third screening, 14 compounds with an average energy score over 65 were selected. Ten compounds that do not violate Lipinski’s rule were identified as final candidates for HGF β-chain inhibitors. The ChemBridge ID, IUPAC name, GOLD score, and chemical structure are shown in Table S1 and Figure S1.

### 2.3. Experimental Validation of HGF Inhibition by Compounds Using pMet ELISA

**Figure 3:**
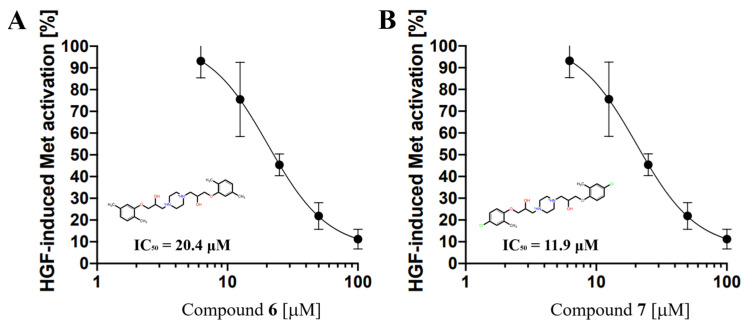
Compound **6** and Compound **7** inhibit HGF-induced Met phosphorylation in a human mesothelioma cell line (EHEMES-1). The vertical axis represents the Met activity in the human mesothelioma cell line. (**A**) Compound **6** [μM]. (**B**) Compound **7** [μM].

The inhibition of HGF activity by compounds (Compound **1**–**10**) identified by hierarchical in silico screening was experimentally verified using the pMet ELISA method [12]. The autophosphorylation level of the Met tyrosine kinase domain was quantified in the EHEMES-1 human mesothelioma cell line stimulated by HGF in the presence of the compounds (Figure S2). Among the 10 compounds, Compound **6** and Compound **7** significantly suppressed Met phosphorylation levels, suggesting that the two compounds inhibit the interaction between the HGF β-chain and the Met sema domain. The 50% inhibitory concentration (IC_50_ value) was determined by examining the dose-dependent effects of Compound **6** and Compound **7**. The IC_50_ values for Compound **6** and Compound **7** were 20.4 μM and 11.9 μM, respectively (Figure 3).

### 2.4. Molecular Dynamics Analysis for the Complex Structures of the HGF β-Chain and Compound ***6**/**7***

**Figure 4:**
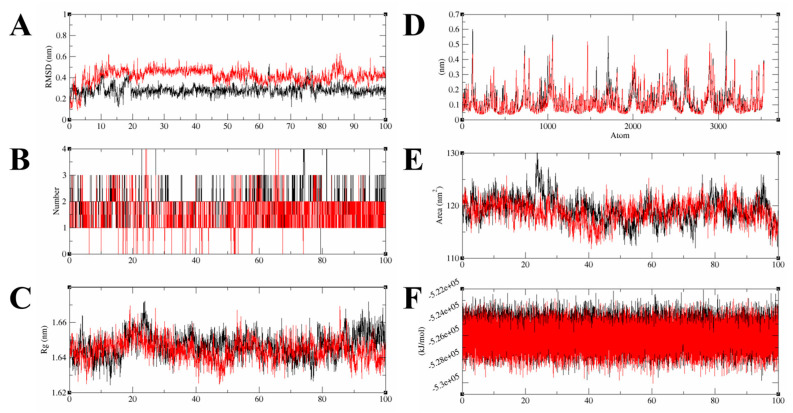
Trajectory analysis of 100 ns MDS of Compound **6**/**7**–HGF β-chain complex. The red and black curves indicate Compound **6**–HGF β-chain complex and Compound **7**–HGF β-chain complex, respectively. (**A**) Ligand RMSD. The vertical axis is ligand RMSD value [nm], and the horizontal axis is time [ns]. (**B**) The number of hydrogen bonds between complexes. The vertical axis is the number of hydrogen bonds in the complex. (**C**) Rg value. The vertical value is Rg value [nm]. (**D**) RMSFs. The vertical axis is the RMSF value [nm]. The horizontal axis is the atomic number of the protein. (**E**) Solvent-accessible surface areas. The vertical axis is the contact areas [nm^2^]. (**F**) The vertical axis represents the Rg value [nm].

**Figure 5:**
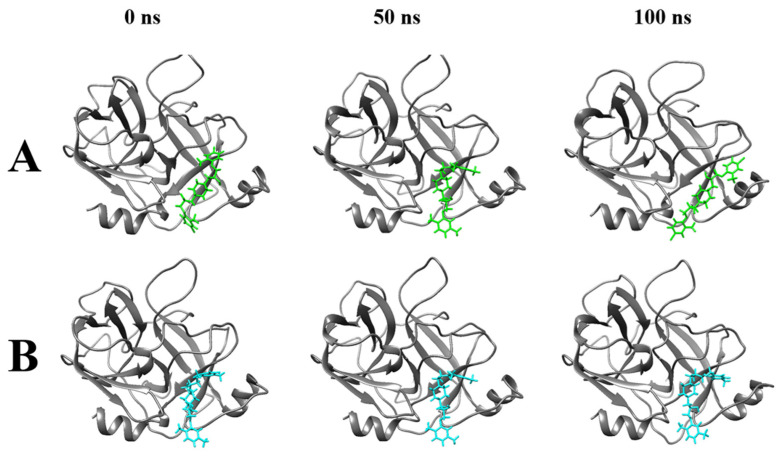
Binding poses of Compound **6** and Compound **7** to the HGF β-chain (0 ns, 50 ns, 100 ns). (**A**) Compound **6**–HGF β-chain. (**B**) Compound **7**–HGF β-chain. Gray ribbons indicate the HGF β-chains, cyan indicates Compound **6**, and green indicates Compound **7**.

**Figure 6:**
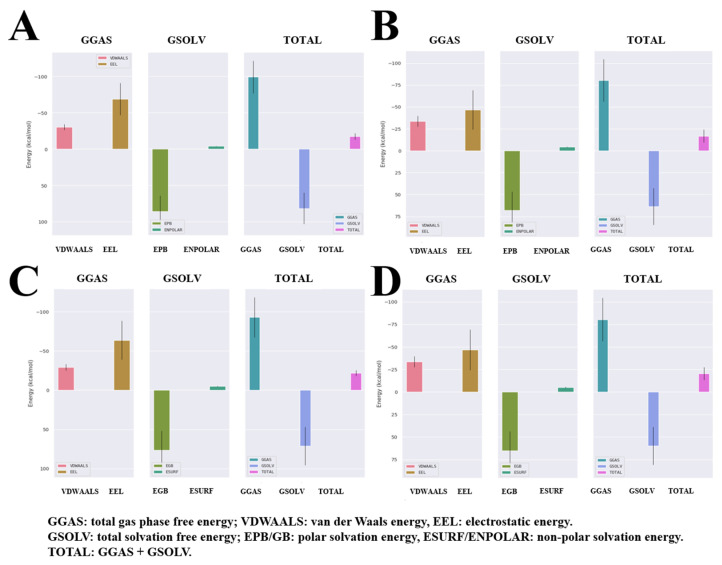
MM-PBSA and MM-GBSA analysis of Compound **6**/**7**–HGF β-chain complex in MDS. (**A**,**B**) ΔG_bind_ values from PB model ((**A**): Compound **6**–HGF β-chain; (**B**): Compound **7**–HGF β-chain). (**C**,**D**) ΔG_bind_ values from GB model ((**C**): Compound **6**–HGF β-chain; (**D**): Compound **7**–HGF β-chain). The vertical axis is the ligand binding energy [kcal/mol]. The horizontal axis is the various energies calculated by MM-PBSA analysis.

Molecular dynamics simulation (MDS) was performed to analyze the time-dependent dynamic changes of the Compound **6**/**7**–HGF β-chain complex structure and to evaluate their binding stability. MDS was performed for 100 ns with the docking pose of the best GOLD score as the initial structure, followed by minimization and equilibration processes. Based on the trajectory data obtained by MDS, the following various interaction modes of the complexes were analyzed. Ligand root mean square deviation (RMSD) indicates the movement of the compound from its original position. The number of hydrogen bonds (Hbond) contributes to the stability of the complex. The radius of gyration (Rg) indicates the folding state of the protein in the system. Root mean square fluctuations (RMSFs) indicate positional changes in the interacting atoms. Ligand solvent-accessible surface areas (SASAs) indicate close binding ability to the protein pocket. Potential energy (PE) indicates the energetic stability of the system. The mean values of ligand RMSD for Compound **6** and Compound **7** were 0.28 ± 0.04 [nm] and 0.42 ± 0.07 [nm], respectively, indicating that Compound **6** and Compound **7** stably bound to the HGF β-chain in MDS for 100 ns (Figure 4A). In 100 ns MDS, Compound **6** and Compound **7** formed at most three stable hydrogen bonds to the HGF β-chain (Figure 4B). The Rg values of Compound **6**/**7**–HGF β-chain complexes were stable around 1.64–1.66 [nm], indicating that the HGF β-chain folding was maintained (Figure 4C). The ligand RMSF values between the Compound **6**–HGF β-chain complex and the Compound **7**–HGF β-chain complex were similar to each other; compound-dependent conformational changes of the HGF β-chain were not observed (Figure 4D). SASA values remained consistently around 120 [nm^2^] throughout the MDS, suggesting a stable compound binding state (Figure 4E). The PE value remained constant around −5.26 × 10^5^ [kJ/mol] in the MDS, indicating system stability (Figure 4F). The complex structures of the Compound **6**/**7**–HGF β-chain in MDS were visualized. Compound **6** and Compound **7** were bound to the binding site of the HGF β-chain with stable conformations for 100 ns of MDS (Figure 5). We calculated the binding free energies (PB and GB models) by the Gmx_MMPBSA method [13,14]. The ΔG_bind_ values for Compound **6** and Compound **7** calculated by the PB model were −17.42 [kcal/mol] and −16.81 [kcal/mol], respectively. The ΔG_bind_ values for Compound **6** and Compound **7** calculated by the GB model were −22.59 [kcal/mol] and −20.41 [kcal/mol], respectively (Figure 6).

### 2.5. Analysis of the Binding Mode of Compound ***6*** and Compound ***7*** to the HGF β-Chains

**Figure 7:**
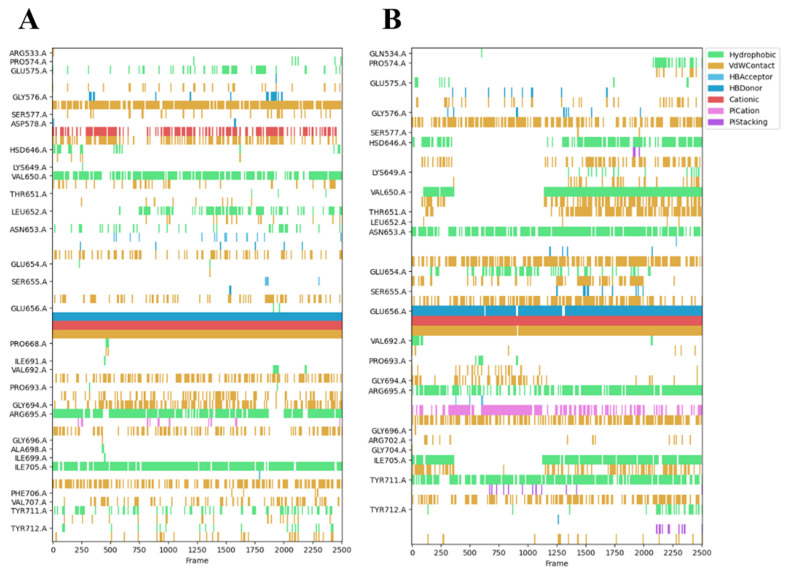
Fingerprint analysis of Compound **6**/**7**–HGF β-chain complex performed on 100 ns MDS trajectory data. (**A**) Compound **6**–HGF β-chain. (**B**) Compound **7**–HGF β-chain.

**Figure 8:**
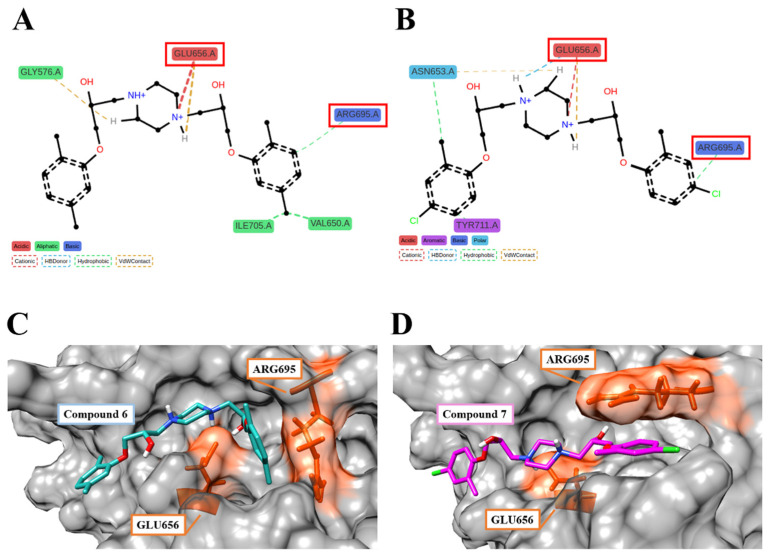
The docking structures for Compound **6**/**7** and the HGF β-chain. (**A**,**B**) Interacting residues between Compound **6**/**7** and the HGF β-chains. Red boxes indicate CRs. (**C**,**D**) Compound **6**/**7** were found to bind in more than 70% of the total simulation periods. The residues highlighted in orange are CRs. ((**A**,**C**): Compound **6**–HGF β-chain. (**B**,**D**): Compound **7**–HGF β-chain).

**Figure 9:**
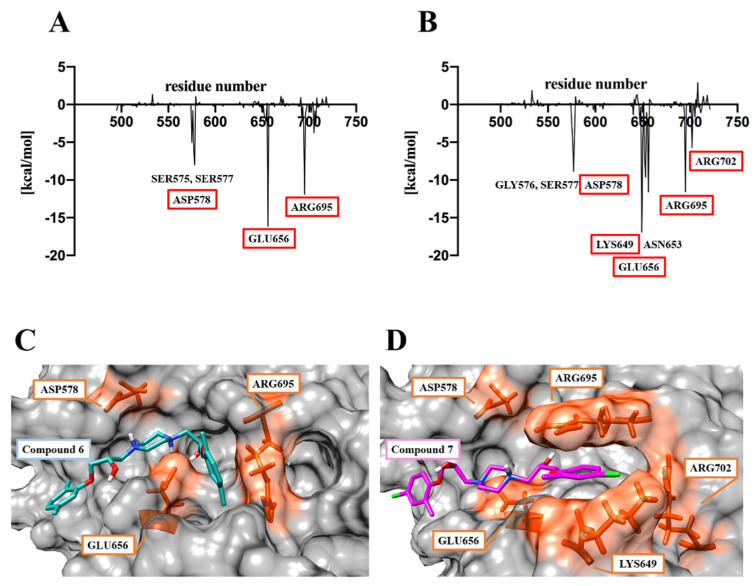
Prediction of interaction residues for Compound **6**/**7** and the HGF β-chain using FMO method. (**A**,**B**) Analysis of IFIE by residue for Compound **6** and Compound **7**. The vertical axis is IFIE [kcal/mol]. The horizontal axis is the residue name of the HGF β-chain. Red boxes indicate CRs. (**C**,**D**) Predicted binding mode of Compound **6**/**7**–HGF β-chain complexes. The residues highlighted in the orange box are CRs. ((**A**,**C**): Compound **6**–HGF β-chain. (**B**,**D**): Compound **7**–HGF β-chain).

Trajectories generated by 100 ns MDS of the Compound **6**/**7**–HGF β-chain complex were analyzed by the fingerprint method [15]. The type of interaction between the amino acid residues of the HGF β-chain and the functional groups of the compounds was predicted. In the 100 ns MDS, Compound **6** and Compound **7** formed interactions with 29 and 24 amino acid residues of the HGF β-chain, respectively (Figure 7A,B). Compound **6** formed interactions with the HGF β-chains GLY576, VAL650, GLU656, ARG695, and ILE705 for longer than 70% of the total simulation duration. The piperazine ring of Compound **6** forms Van der Waals and π–cation interactions with GLY576 and GLU656, respectively, while the phenyl group forms hydrophobic interactions with VAL650, ARG695, and ILE705. Compound **7** forms interactions with the HGF β-chains ASN653, GLU656, ARG695, and TYR711 for longer than 70% of the total simulation duration. The piperazine ring of Compound **7** forms van der Waals interactions, π–cation interactions, and hydrogen bonds with ASN653 and GLU656, respectively. The chlorophenyl groups at both ends form hydrophobic interactions with ASN653, ARG695, and TYR711, respectively. The piperazine ring of Compound **6** and Compound **7** forms van der Waals, π–cation interactions, and electrostatic interactions with GLU656. GLU656 and ARG695 have been identified as critical residues (CRs) mediating the interaction between the HGF β-chain and the Met sema domain. (Figure 8A–D) [5]. The fragment molecular orbital (FMO) method was used to calculate the inter-fragment interaction energy (IFIE) between Compound **6**/**7** and the HGF β-chain. The IFIE_total_ (sum of IFIEs) for Compound **6** and Compound **7** was −61.48 [kcal/mol] and −85.67 [kcal/mol], respectively [16,17]. Residue-specific IFIE analysis confirmed potent interactions of Compound **6** and Compound **7** with SER577, ASP578, GLU656, and ARG695. Molecular dynamics and quantum mechanical analysis of the interacting amino acid residues suggest the involvement of ARG695/GLU656 in intermolecular bond formation. ASP578, LYS649, GLU656, ARG695, and ARG702 are also CRs (Figure 9A–D). Compound **6** and Compound **7** were postulated to bind to these amino acid residues, thereby competitively inhibiting Met in the interface pocket of the HGF β-chain.

### 2.6. ADME–Tox Predictions for Compound ***6*** and Compound ***7***

Predictions of pharmacokinetic, drug-likeness, and pharmacological affinity properties were performed using the Swiss ADME web server [18]. Although Compound **6** and Compound **7** exceeded the appropriate values for the flexibility parameter in the six Swiss ADME indices (lipophilicity, molecular weight, solubility, saturation, and flexibility), they were determined to be appropriate in the Lipinski, Veber, and Muegge drug-likeness indices. The high gastrointestinal (GI) absorption values of Compound **6** and Compound **7** suggest their suitability for oral administration (Figure S3). We investigated the toxicity of the compounds using the toxicity prediction models (17 models) of the ProTox-III web server [19]. The results showed Compound **6** and Compound **7** toxicities with 0.54–0.99 probability. Compound **6** and Compound **7** were classified as toxicity class 4 (300 < LD_50_ [mg/kg] ≤ 2000) (Figure S4), and the predicted results from Swiss ADME and ProTox-III suggest that structural information of Compound **6** and Compound **7** is likely to be useful for drug development.

### 2.7. Structure and Activity Relationships of Compound ***6*** and Compound ***7*** Analogs

**Figure 10:**
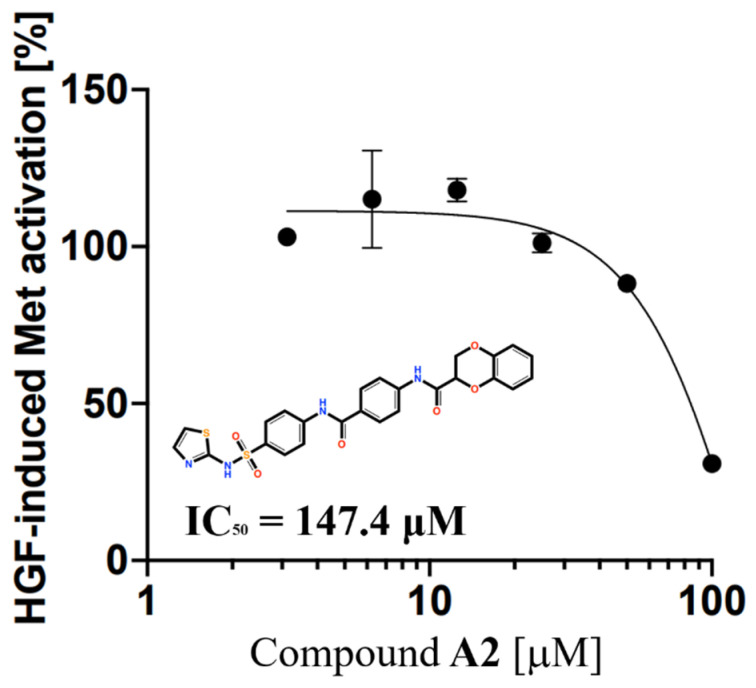
Compound **A2** inhibits HGF-induced Met phosphorylation in EHEMES-1. The vertical axis represents the Met activity in the human mesothelioma cell line.

We searched for Compound **6** and Compound **7** analogs using the Hit2Lead database containing approximately 1.3 million compounds. Five candidates (Compound **A1**–**5** (Table S2, Figures S5 and S6)) were selected using the same screening protocol as in the current study. Subsequent assays using the identical experimental system revealed an IC_50_ value of 147.4 μM for Compound **A2** (Figure 10). However, none of the analogs exhibited improved activity compared to the original Compound **6** and Compound **7**.

## 3. Materials and Methods

### 3.1. The HGF β Structure 

The hepatocyte growth factor (HGF) β-chain was used as the target protein for the in silico SBDS. The crystal structure data of the HGF β-chain (PDBID: 4K3J, resolution: 2.80 Å) were obtained from the RCSB Protein Data Bank (PDB). The Met sema domain, onartuzumab, and ligand (NAG) were deleted from the c-Met and the HGF β-chain complex structure data (4K3J). Hydrogen atoms and partial charges were added to the 3D structure of the HGF β-chain using MOE software [20] and energy minimization calculations for the HGF β-chain were carried out. The DMS [21] and the sphgen programs of DOCK [22] performed binding molecule surface extraction and binding pocket search on the binding interface with the Met sema domain, respectively.

### 3.2. 3D Chemical Structure Library for SBDS

The 3D structure library (Chembridge, 154,118 compounds) used in the in silico SBDS was obtained from the web database of Ressource Parisienne en BioInformatique Structurale (RPBS) [23]. This compound library is processed with the ADME–Tox (absorption, distribution, metabolism, excretion, toxicity) filter.

### 3.3. Hierarchical In Silico SBDS

Rigid docking with DOCK was performed for the first screening in the hierarchical in silico SBDS. Flexible docking simulations with GOLD based on genetic algorithms were performed for the second and third screening. In the second screening, compounds were selected using the energy score of a single conformer with GOLD. In the third screening, compounds were selected using the energy scores of 10 conformers per compound with GOLD. In our fourth screening, we selected compounds that met all five of Lipinski’s rules.

### 3.4. pMet ELISA

EHEMES-1 cells (10,000 cells/well) were cultured in 96-well plates with RPMI 1640 medium (FUJIFILM Wako Pure Chemical Corporation, Osaka, Japan) supplemented with supplemented with 10% fetal bovine serum and 2 mM L-glutamine at 37 °C, 5% CO_2_ for 24 h. Cells were stimulated with HGF for 10 min, with or without compounds. After fixation in 4% paraformaldehyde, cells were permeabilized and blocked with 5% goat serum and 0.02% Triton X-100 (NACALAI TESQE, INC., Kyoto, Japan) in phosphate-buffered saline (PBS) for 30 min. Cells were incubated with phospho-Met (Tyr1234/1235) XP rabbit mAb (Cell Signaling Technology, Danvers, MA, USA) for 2 h, and washed with PBS three times, followed by horseradish peroxidase-conjugated anti-rabbit goat antibody (Dako, CA, USA) for 1 h. After washing, chemiluminescence was developed using ImmunoStar LD reagent (FUJIFILM Wako Pure Chemical Corporation, Osaka, Japan) and measured with an ARVO MX luminometer (PerkinElmer, Waltham, MA, USA).

### 3.5. Molecular Dynamics Simulation

To evaluate the binding stability of Compound **6** and Compound **7** to the HGF β-chain, MDS with the GROMACS 2023.2 [24,25] package was performed. We built the cubic MDS system using the CHARMM-GUI web server Solution Builder [26,27,28]. We have included protein–compound complexes in the MDS system, with additional water molecules and ions. The cubic MDS system was solvated by TIP3P water molecule models. The system was neutralized with Na^+^ and Cl^−^ ions at a salt concentration of 0.13 M. We used the CHARMM36m [29] force field and the LINCS algorithm [30], and we used the particle mesh Ewald method [31] for the long-range electrostatic interactions. The created systems were energy-minimized in up to 5000 steps by the steepest descent method. The first- and second-step equilibrations were performed under NVT (310 K) and NPT (310 K, 1 bar) conditions, respectively. Position-unconstrained production MDS was carried out under NPT conditions (2fs time step). The 100 ns MDS was performed three times. After the 100 ns MDS calculations, the ligand RMSD values were analyzed. The binding free energies between Compound **6** and Compound **7** and the HGF β-chain were calculated by molecular mechanics Poisson–Boltzmann surface area (MM-PBSA) and molecular mechanics generalized Born surface area (MM-GBSA) analysis [32]. The rms module of GROMACS was used to calculate ligand RMSD values, and the Gmx_MMPBSA tool was used to calculate binding free energies in MM-PBSA and MM-GBSA analysis. 

### 3.6. MDS Trajectory Data Analysis

MDS trajectory data were used to analyze the binding stability of Compound **6**/**7** to the HGF β-chain. The sasa module of GROMACS was used to calculate the solvent contact area of the compounds. The radius of gyration of the protein–ligand complex was calculated using the gyrate module. The number of hydrogen bonds between proteins/compounds was calculated using the hbond module. Fingerprinting analysis was performed using 100 ns MDS trajectory data by ProLIF.

### 3.7. Ab Initio FMO Calculations

Ab initio FMO calculations were performed by ABINIT-MP6.0 [33]. The Compound **6**/**7**–HGF β-chain complex structure obtained from the 100 ns MDS was optimized for complex structure and charge state by the MOE structure preparation module. The complex structures were automatically fragmented by the abinit-mp program. The theoretical level in the FMO calculation was set to MP2/6-31G. After FMO calculation, IFIE and IFIE_total_ were calculated using BioStation Viewer 15.0 [34]. 

### 3.8. Prediction of ADME–Tox

The ADME–Tox properties of Compound **6** and Compound **7** were predicted using Swiss ADME and ProTox-III. 

## 4. Conclusions

Hierarchical in silico SBDS targeting the HGF β-chain identified novel Compound **6** and Compound **7** that bind to the HGF β-chain and have the potential to inhibit HGF binding to Met. The IC_50_ values of Compound **6** and Compound **7** were 20.4 μM and 11.9 μM, respectively. The binding free energies predicted by MDS and MM-PBSA, MM-GBSA, and FMO methods suggest that Compound **6** and Compound **7** stably bind to the interface pocket of the HGF β-chain for the Met sema domain binding. Compound **6** and Compound **7** were found to bind potently to GLU656 and ARG695. Compound **6** and Compound **7** are expected to be breakthrough inhibitors with a novel backbone and mechanism of action that target HGF and inhibit the HGF/Met interaction and are promising lead compounds in the development of small-molecule anticancer drugs. By searching for analogs of Compound **6** and Compound **7** and modifying them through structure–activity relationship studies and organic synthesis, it is expected to develop anticancer agents with higher inhibitory activity against HGF.

## Data Availability

The data presented in this study are available upon request from the corresponding authors.

## Supplementary Materials

The following supporting information can be downloaded at https://www.mdpi.com/article/10.3390/molecules30081801/s1: Table S1: Characteristics of candidate compounds and GOLD score; Figure S1: Chemical structure of candidate compounds; Figure S2: Effects of Compounds **1**–**10** on cellular Met phosphorylation levels. The vertical axis is Met activation [%]; Figure S3: Prediction of Compound **6** and Compound **7** pharmacokinetic properties by the Swiss ADME web server; Figure S4: Toxicity prediction of Compound **6** and Compound **7** by the ProTox-III web server; Table S2: Characteristics of analog compounds and GOLD score; Figure S5: Chemical structure of analogous compounds; Figure S6: Effects of analog Compounds **A1**–**5** on cellular Met phosphorylation levels.
