# Supplementary material for: Identification of Novel Compounds That Bind to the HGF β-Chain In Silico, Verification by Molecular Mechanics and Quantum Mechanics, and Validation of Their HGF Inhibitory Activity In Vitro
Source: Molecules. 2025 Apr 17;30(8):1801. doi: 10.3390/molecules30081801 (PMC12029800; doi:10.3390/molecules30081801)
Supplement: Supplementary file 1 [file molecules-30-01801-s001.zip › molecules-3510940-supplementary.pdf]

Table S1. Characteristics of candidate compounds and GOLD score.

Name	ID	IUPAC	GOLD score
Compound **1**	5280580	bis[(benzylamino)methyl]phosphinic acid hydrochloride	67.6
Compound **2**	6679566	dimethyl 5,5'-[1,4-piperazinediylbis(methylene)]di(2-furoate)	66.9
Compound **3**	6702762	5-(3,4-dichlorophenyl)-3-hydroxy-4-(4-methoxybenzoyl)-1-[2-(1-piperazinyl)ethyl]-1,5-dihydro-2H-pyrrol-2-one	69.4
Compound **4**	6727276	5-(3,4-dimethoxyphenyl)-3-hydroxy-4-(4-methoxybenzoyl)-1-[2-(1-piperazinyl)ethyl]-1,5-dihydro-2H-pyrrol-2-one	65.3
Compound **5**	6942787	6-ethyl-7-hydroxy-8-{[4-(2-hydroxyethyl)-1-piperazinyl]methyl}-3-(1-methyl-1H-benzimidazol-2-yl)-4H-chromen-4-one	65.6
Compound **6**	7384989	1,1'-(1,4-piperazinediyl)bis[3-(2,5-dimethylphenoxy)-2-propanol]	67.0
Compound **7**	7396269	1,1'-(1,4-piperazinediyl)bis[3-(4-chloro-2-methylphenoxy)-2-propanol]	66.3
Compound **8**	7421480	N,N'-bis(3,4-dimethoxybenzyl)-1,2-propanediamine dihydrochloride	70.4
Compound **9**	7888750	2-({5-[(3,4-dichlorophenoxy)methyl]-4-phenyl-4H-1,2,4-triazol-3-yl}thio)-N-(1,3-dimethyl-2,6-dioxo-1,2,3,6-tetrahydro-4-pyrimidinyl)acetamide	69.3
Compound **10**	7918409	4,4'-[1,2-propanediylbis(iminomethylene)]bis(2-methoxyphenol) hydrochloride	68.2

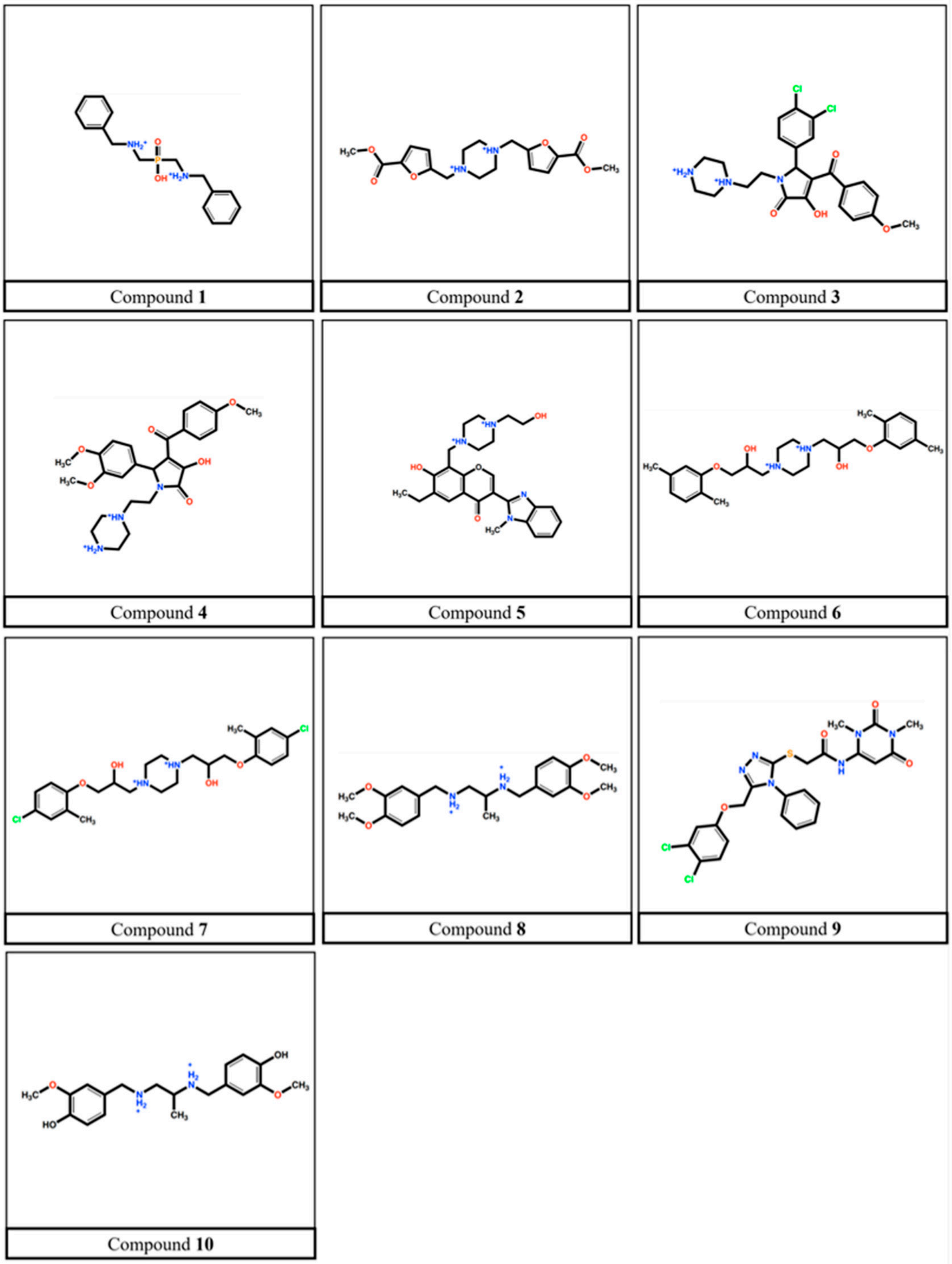

Figure S1. Chemical structure of candidate compounds.

The amino group was identified to be protonated, resulting in a positively charged structure.

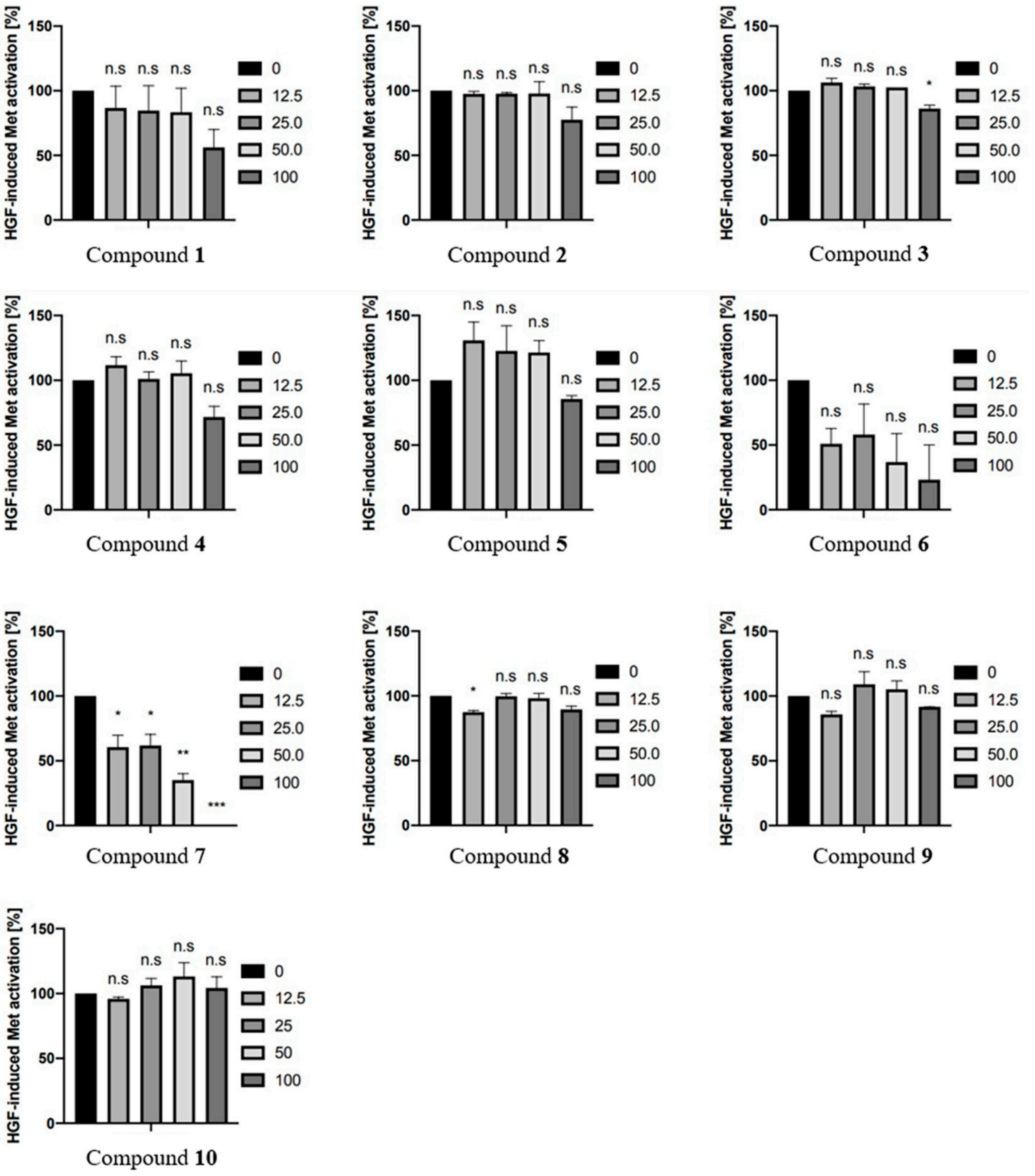

Figure S2. Effects of Compounds **1-10** on cellular Met phosphorylation levels. The vertical axis is Met activation [%]. The bars indicate the concentration of the compounds, respectively. The legends means 0 μM, 12.5 μM, 25.0 μM, 50 μM, and 100 μM from the top to the bottom. $p < 0.032$ (*), 0.0021 (**), 0.0002 (***), 0.0001 (****).

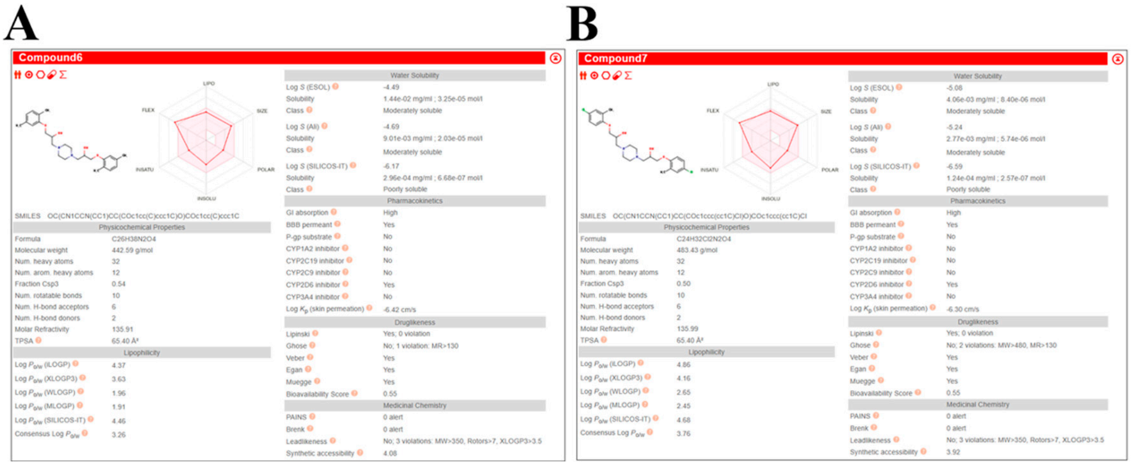

Figure S3. Prediction of Compound **6** and **7** pharmacokinetic properties by the Swiss ADME web server. (A) Compound **6.** (B) Compound **7**.

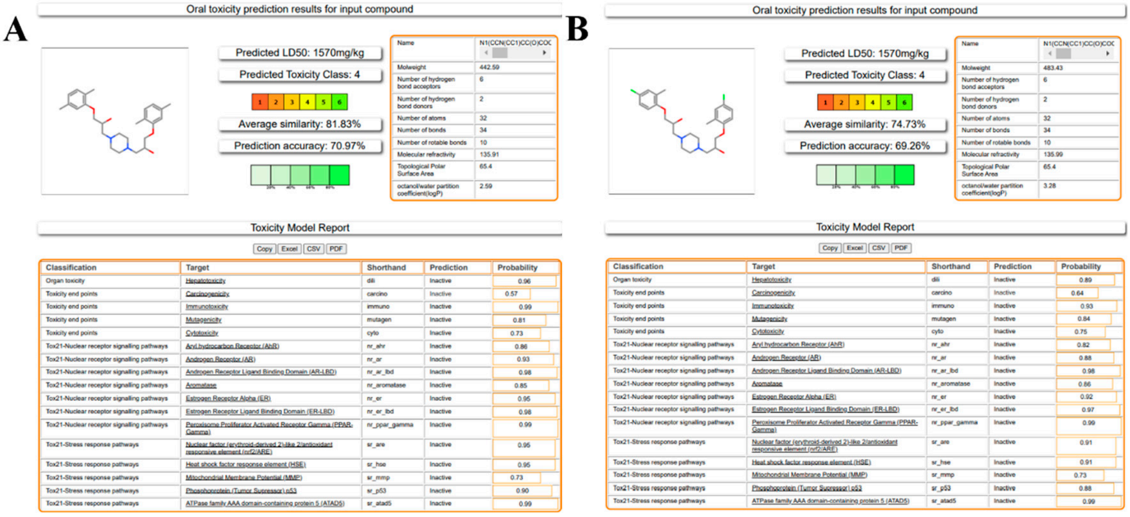

Figure S4. Toxicity prediction of Compound **6** and **7** by the ProTox-III web server. (A) Compound **6.** (B) Compound **7**.

Table S2. Characteristics of analogue compounds and GOLD score.

Name	ID	IUPAC	GOLD score
Compound **A1**	5493793	potassium {3-[[3-(4-chloro-3-methylphenoxy)-2-hydroxypropyl](methyl)amino]-2-hydroxypropyl}sulfamate	65.7
Compound **A2**	7727645	N-{4-[({4-[(1,3-thiazol-2-ylamino)sulfonyl]phenyl}amino)carbonyl]phenyl}-2,3-dihydro-1,4-benzodioxine-2-carboxamide	65.2
Compound **A3**	17859237	1-[3-({[(2-methylimidazo[2,1-b][1,3,4]thiadiazol-6-yl)methyl]amino}methyl)phenoxy]-3-(4-morpholinyl)-2-propanol	65.8
Compound **A4**	23174808	2-{4-(1,3-benzothiazol-2-yl)-2-[(dimethylamino)methyl]phenoxy}-N-(2,3-dihydro-1,4-benzodioxin-2-ylmethyl)acetamide	73.6
Compound **A5**	30256486	4-[(2,3-dihydro-1,4-benzodioxin-2-ylmethyl)amino]-5-methyl-N-[3-(4-morpholinyl)propyl]thieno[2,3-d]pyrimidine-6-carboxamide	65.2

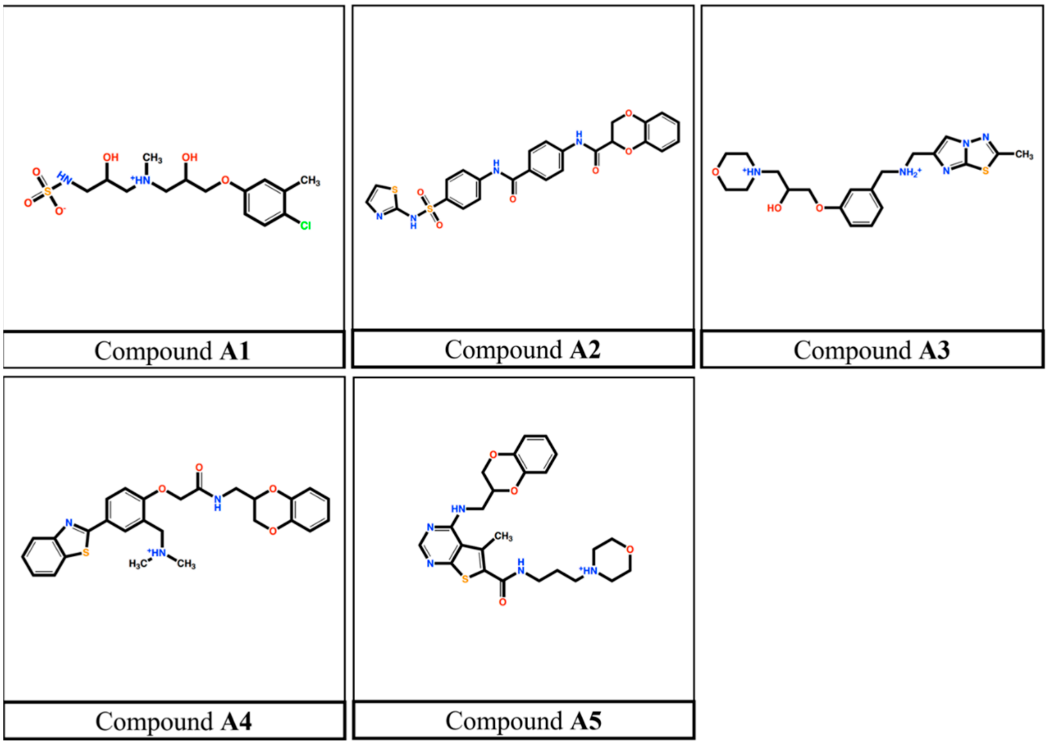

Figure S5. Chemical structure of analogous compounds.

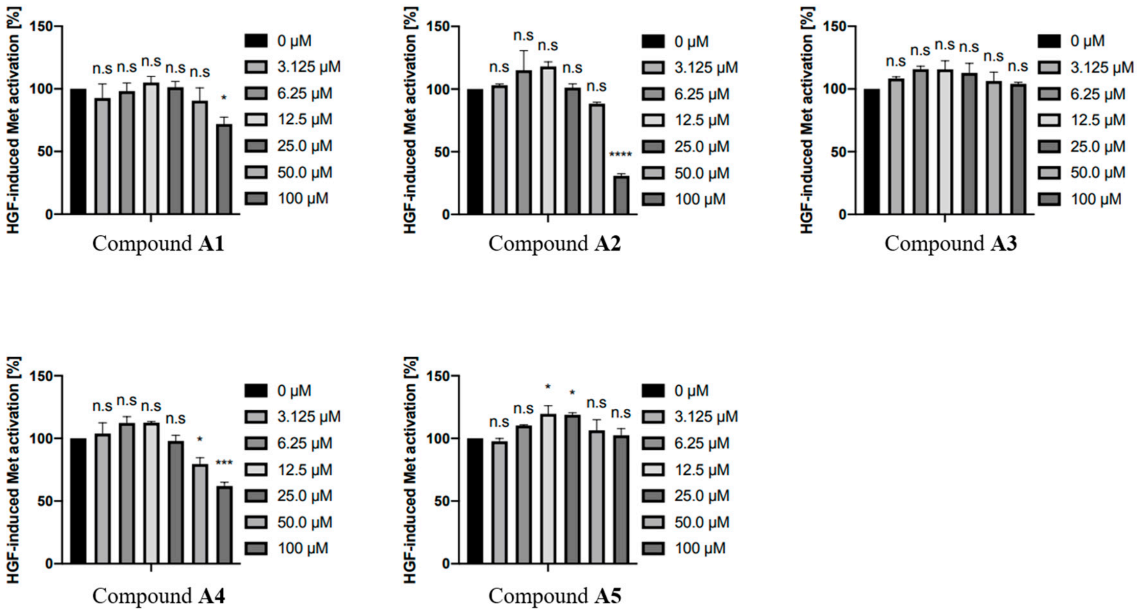

Figure S6. Effects of analogue Compounds A**1-5** on cellular Met phosphorylation levels. The vertical axis is Met activation [%]. The bars indicate the concentration of the compounds, respectively. The legends means 0 μM, 3.125 μM, 6.25 μM, 12.5 μM, 25.0 μM, 50.0 μM and 100 μM from the top to the bottom. $p < 0.032$ (*), 0.0021 (**), 0.0002 (***), 0.0001 (****).
